# Hyperventilation Syndrome and Hypocalcemia: A Unique Case in Autism Spectrum Disorder

**DOI:** 10.7759/cureus.59639

**Published:** 2024-05-04

**Authors:** Rahul Kamboj, Ajay Singh, Deepthi Ketha, Arghadip Das, Sachin M Chaudhary, Hadeeqa Idris, Mohitha C Mallipaddi

**Affiliations:** 1 Psychiatry and Behavioral Sciences, King George's Medical University, Lucknow, IND; 2 Internal Medicine, Shri Ram Murti Smarak Institute of Medical Sciences, Bareilly, IND; 3 Internal Medicine, Anam Chenchu Subba Reddy (ACSR) Government Medical College, Nellore, IND; 4 Internal Medicine, Nil Ratan Sircar Medical College and Hospital, Kolkata, IND; 5 Internal Medicine, Gujarat Cancer Society (GCS) Medical College, Hospital, and Research Centre, Ahmedabad, IND; 6 Internal Medicine, Shifa International Hospital, Islamabad, PAK; 7 Internal Medicine, Sri Venkateswara Institute of Medical Sciences, Visakhapatnam, IND

**Keywords:** anxiety management, respiratory alkalosis, panic attacks, autism spectrum disorder, hypocalcemia, hyperventilation syndrome

## Abstract

This case report delves into the rare occurrence of hyperventilation syndrome (HVS) with hypocalcemia in an 18-year-old female diagnosed with autism spectrum disorder (ASD). The rare occurrence highlights the importance of recognizing the potential association between HVS, hypocalcemia, and ASD, emphasizing the need for comprehensive evaluation and management strategies in individuals with ASD presenting with unusual symptoms. Despite ongoing psychotherapeutic treatment, the patient's clinical examination revealed ASD-related communication anomalies. Treatment with Escitalopram resolved panic attacks but left residual anxiety. During an emergency room visit for menstrual-related abdominal pain, a hyperventilation crisis ensued, leading to respiratory alkalosis and hypocalcemia. Swift intervention, including closed mask ventilation and electrolyte infusion, successfully alleviated symptoms. Follow-up assessments indicated normal thyroid function and vitamin D levels. The case highlights the necessity for clinicians to consider electrolyte imbalances in anxiety attacks among ASD patients, emphasizing the importance of timely management for patient safety. The intricate interplay between hyperventilation syndrome, anxiety, and hypocalcemia in ASD patients is explored, offering valuable insights for the nuanced understanding and comprehensive assessment of such cases.

## Introduction

Hyperventilation syndrome (HVS), characterized by rapid or deep breathing resulting in reduced carbon dioxide levels in the blood, is often linked with anxiety and panic disorder [1]. This disorder is marked by sudden and recurrent anxiety or panic attacks, presenting symptoms like palpitations, sweating, shortness of breath, chest pain, and more [1]. These intense episodes of fear and discomfort are diagnosed as an anxiety disorder when accompanied by persistent concern, worrying about consequences, or behavioral changes related to the attack. Notably, individuals with autism spectrum disorders (ASD) are considered to be at a higher risk of experiencing these anxiety or panic attacks, further complicating the relationship between HVS and mental health challenges in individuals with ASD [2]. Every child or adult with ASD has a distinctive profile, making ASD a heterogeneous condition [3]. According to global estimates, 1 in 132 people have ASD [4]. People with ASD frequently have additional disorders, including mood swings and anxiety, as a result of these difficulties with social functioning and communication [3]. Co-morbidities are thought to affect up to 72% of children with ASD [5]. Nevertheless, because co-morbid illnesses can be challenging to detect, co-morbidity statistics for ASD are still largely unclear [6-7]. With rates ranging from 40 to 50%, anxiety and depression are two of the most prevalent co-morbidities associated with ASD [8]. Individuals with ASD frequently lack the ability to convey their feelings of stress or frustration because of communication issues. It is difficult to determine the best course of action when co-morbidity symptoms overlap and each child has distinct symptoms. The difficulties in communicating and the distinctiveness of symptoms make it challenging to arrive at the right diagnosis or select the best course of treatment [7]. As a result, an in-depth understanding of each condition provides valuable insights into their intricate interplay and sheds light on the potential impact on the overall well-being of individuals with autism. We present a rare case of respiratory alkalosis, which induced secondary hypocalcemia during an anxiety attack in a female adolescent with ASD. The present case study explores the recognition of rarely reported secondary conditions associated with anxiety symptoms and aims to provide suggestions for the critical assessment of anxiety in individuals with autism and intellectual disability (ID).

## Case presentation

An 18-year-old female sought clinic evaluation earlier this year due to persistent panic attacks that significantly impaired her daily functioning despite undergoing ongoing private psychotherapeutic treatment for the past six months. Referred from the emergency room, her clinical examination revealed an androgynous appearance, a vaguely modest posture, and a depressed mood. She exhibited poor eye contact, constricted affect, and speech hesitant in style and limited in verbal output, requiring redirection throughout the encounter. Looseness of associations was noted, though cognitive function remained intact.

The patient presented with symptoms of anxiety with expressed triggers by school duties and expectations; her anxiety occasionally escalated into panic attack-type symptoms. Additional communication anomalies and social interaction issues, along with a long-standing habit of "posing," were identified. In addition to her psychiatric condition, a subsequent diagnosis of High Functioning Autism Spectrum Disorder was made, surprising as she was not previously considered for this disorder.

Treatment with escitalopram 5 mg PO for six weeks resolved panic attacks and improved mood, yet anxiety persisted, especially in unexpected or challenging social situations. During an emergency room visit for severe abdominal pain (which was related to the menstrual cycle), anxiety about potential surgery arose. A hyperventilation crisis occurred, leading to respiratory alkalosis, characterized by a decrease in blood carbon dioxide levels and an increase in blood pH. This alkalosis resulted in hypocalcemia, as respiratory alkalosis lead to a shift of ionized calcium into cells, causing a decrease in blood calcium levels. There was sweating and paresthesia due to the underlying anxiety, exacerbating the symptoms of hypocalcemia.

Pulmonary function tests were negative for asthma. Closed mask ventilation using air/oxygen (1.5/1.5 L) per minute for one minute was used to control hyperventilation. Ten percent calcium gluconate in 10 milliliters (ml) was infused slowly over five minutes, and KCl 10 milliequivalents (meq) diluted in 50 ml normal saline solution was given over 30 minutes to address the electrolyte imbalance that was the primary cause of the symptoms and signs of HVS. Somatic symptoms, such as perioral numbness, paresthesia in both hands, finger stiffness, nausea, and headaches, gradually resolved once the patient's hyperventilation reduced and the intravenous electrolyte infusion was finished. Following the patient's return to the recovery room, a repeat arterial blood gas analysis (ABGA) revealed pH 7.41, PaCO2 48 mmHg, and PaO2 72 mmHg, indicating better alkalosis; also, there was an increase in potassium concentration of 2.9 mmol/L and a modest elevation in ionized calcium of 0.95 mmol/L. The patient was discharged on the fifth day with analgesics, instructions for serum calcium/phosphorus balance testing, and assessments for thyroid function, parathyroid hormone, calcitonin, and vitamin D. All these test results were reported in normal ranges.

Post-hyperventilation episode, the psychiatry team was consulted for the re-evaluation of the patient's psychiatric disorder. Reassurance, psychoeducation, cognitive behavioral therapy, and initiation of lorazepam 1 mg treatment (for four months) followed.

## Discussion

**Figure 1:**
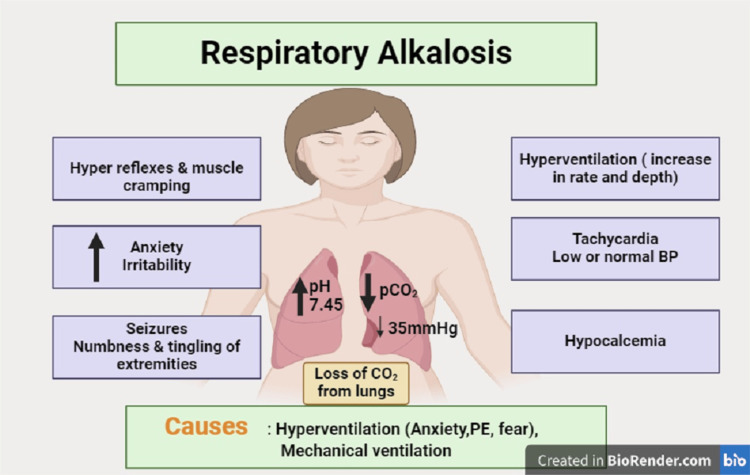
Pathophysiology of hypocalcemia due to hyperventilation Acute hypocapnia (partial pressure of arterial carbon dioxide (PaCO2) less than 35 mmHg) causes a reduction of serum levels of potassium and phosphate secondary to increased intracellular shifts of these ions. A reduction in free serum calcium also occurs. Calcium reduction is secondary to increased binding of calcium to serum albumin due to the change in pH. Many of the symptoms present in persons with respiratory alkalosis are related to hypocalcemia BP - blood pressure, PE - pulmonary embolism Image credits: Rahul Kamboj and Deepthi Ketha

Hypocalcemia can develop due to various mechanisms, including impaired parathyroid hormone secretion or action, inadequate vitamin D synthesis or absorption, renal failure leading to decreased calcium reabsorption, and acute respiratory alkalosis causing a shift of ionized calcium into cells. Hypocalcemia due to hyperventilation is a condition wherein a decline in the partial pressure of CO2 induces respiratory alkalosis (Figure 1) [9]. This alteration results in the constriction of cerebral circulation, thereby precipitating primary neurological symptoms such as confusion, dizziness, and vertigo [10]. Subsequently, an electrolyte imbalance ensues: in the context of alkalosis, blood calcium forms complexes with proteins, leading to transient hypocalcemia, hypokalemia, and hypomagnesemia [11]. The impairment of cellular calcium absorption gives rise to manifestations including spasms, cramps, and involuntary dystonic muscle movements. Excessive hyperventilation during stressful, fearful, or anxious conditions can trigger the HVS syndrome, which results in hypocapnia and the related systemic symptoms and indications [12]. There is a recognized wide variety of HVS symptoms, and each patient experiences symptoms in a different way. Sweating, central nervous system symptoms, tingling in the hand and numbness in the feet, and others might appear during and after hyperventilation [2]. Psychological HVS can also be primarily caused by air hunger combined with fast breathing brought on by worry [12]. Asthma, chronic bronchitis, emphysema, and chronic obstructive pulmonary disease (COPD) are among the respiratory and organic causes of hyperventilation. Pulmonary embolism, encephalitis, brain tumors, hyperparathyroidism, and ASD are less common causes [13].

In this instance, there was no history of chronic obstructive pulmonary disease (COPD), no aberrant results from a pulmonary function test, and a history of pulmonary embolism, which is typically linked to elevated carbon dioxide levels, hypoxia, and chest discomfort. As a result, we did not believe that the patient's HVS had an organic etiology. Hyperventilation can physiologically happen during a woman's menstrual cycle. Growing progesterone activates the cervical carotid body and central nervous system during the second half of the menstrual cycle, causing physiological hyperventilation [13]. Besides ASD, this was the correlated factor contributing to hyperventilation in our patient, stemming from anxiety and psychological trauma provoked by menstrual abdominal cramps.

The use of closed mask ventilation, also known as a rebreathing bag, is a recognized respiratory intervention for managing rapidly progressing hypocapnia in HVS patients [14]. However, it is important to exercise extra caution when performing closed mask ventilation in hypoxic patients or in patients who already have lung conditions. For patients with palpitations, sweating, or trembling, drug therapy for HVS involves the use of beta-blockers; however, patients with COPD who are at risk of broncho-obstructive disorders should use caution while using these medications. The use of sedatives (benzodiazepines) may be taken into consideration to reduce anxiety or sadness [15].

In the realm of high-functioning autism spectrum disorders, it is widely acknowledged that affective and emotional crises are markedly prevalent, with a substantial percentage of individuals experiencing them. Cognitive-behavioral therapy has demonstrated notable efficacy, resulting in a significant reduction in symptoms [2, 16]. Key therapeutic objectives encompass the acquisition of emotional recognition skills, the challenging of anxious thoughts, the cultivation of discomfort tolerance, and the confrontation of anxious symptoms [17]. It is plausible that in the case of this particular patient, the anxiety level was notably elevated.

HVS with hypocalcemia in the context of an anxiety crisis has rarely been reported before.

## Conclusions

Conclusively, we identified and treated HVS triggered by an anxiety attack in a patient with ASD. The presentation included symptoms and signs of hypocalcemia induced by hyperventilation during the anxiety attack. Physicians should remain vigilant about the potential for electrolyte imbalance caused by HVS during anxiety attacks. The inclusion of a psychiatric team would benefit the patient's prognosis of care due to the complexities ASD provides. Hence, timely and proper management of HVS is imperative to ensure patient safety.
